# Microarray-based approach identifies microRNAs and their target functional patterns in polycystic kidney disease

**DOI:** 10.1186/1471-2164-9-624

**Published:** 2008-12-23

**Authors:** Priyanka Pandey, Benedikt Brors, Prashant K Srivastava, Andrea Bott, Susanne NE Boehn, Herrmann-Josef Groene, Norbert Gretz

**Affiliations:** 1 Medical Research Center, University Hospital Mannheim, D-68167 Mannheim, Germany; 2 Department of Theoretical Bioinformatics, German Cancer Research Center, D-69120 Heidelberg, Germany; 3 Department of Cellular and Molecular Pathology, German Cancer Research Center, D-69120 Heidelberg, Germany

## Abstract

**Background:**

MicroRNAs (miRNAs) play key roles in mammalian gene expression and several cellular processes, including differentiation, development, apoptosis and cancer pathomechanisms. Recently the biological importance of primary cilia has been recognized in a number of human genetic diseases. Numerous disorders are related to cilia dysfunction, including polycystic kidney disease (PKD). Although involvement of certain genes and transcriptional networks in PKD development has been shown, not much is known how they are regulated molecularly.

**Results:**

Given the emerging role of miRNAs in gene expression, we explored the possibilities of miRNA-based regulations in PKD. Here, we analyzed the simultaneous expression changes of miRNAs and mRNAs by microarrays. 935 genes, classified into 24 functional categories, were differentially regulated between PKD and control animals. In parallel, 30 miRNAs were differentially regulated in PKD rats: our results suggest that several miRNAs might be involved in regulating genetic switches in PKD. Furthermore, we describe some newly detected miRNAs, miR-31 and miR-217, in the kidney which have not been reported previously. We determine functionally related gene sets, or pathways to reveal the functional correlation between differentially expressed mRNAs and miRNAs.

**Conclusion:**

We find that the functional patterns of predicted miRNA targets and differentially expressed mRNAs are similar. Our results suggest an important role of miRNAs in specific pathways underlying PKD.

## Background

MicroRNAs (miRNAs) are known to regulate the expression of key genes relevant to cancer and potentially other diseases [1-3]. MiRNAs are short noncoding RNAs of about 22 nt length that have recently been shown to play important roles in mammalian gene expression [1,3-5]. They induce posttranscriptional gene repression by blocking protein translation (by binding to the 3' UTR of their target genes) or by inducing mRNA degradation, and have the potential to play central roles in physiological and pathological conditions. Recently, it has been shown that miRNAs can also increase translation [6,7]. The physiological conditions of a cell seem to affect the recruitment of regulatory proteins, which can alter the effect of a miRNA.

MiRNAs are transcribed as long primary transcripts (pri-miRNAs), some of them being polycistronic, which are processed in the cell nucleus by an enzyme called Drosha, yielding precursor miRNAs (pre-miRNAs) that exhibit a characteristic stem-loop sequence [8]. These are exported into the cytosol where mature miRNAs are generated by the RNAse Dicer, producing the small single-stranded miRNA [8]. Translational inhibition, which seems to be the major mode of action in animals, is performed by a riboprotein complex called RNA-induced silencing complex (RISC) consisting of the miRNA and proteins of the argonaute family [9,10].

MiRNAs are involved in several cellular processes, including cellular differentiation [11,12], organism development [13,14], and apoptosis [15,16]. While all of these are conserved in metazoans, the number of conserved miRNAs between mammals suggests that there are additional functions only found in vertebrates [4], e.g. controlling hematopoietic differentiation [17]. Recent studies provide growing evidence for the involvement of miRNAs in cancer pathomechanisms [18-21]. However, to date nothing is known regarding miRNAs in the context of Polycystic Kidney Diseases.

Cilia and flagella are ancient, evolutionary conserved organelles that project from cell surfaces to perform diverse biological roles, including whole-cell locomotion, movement of fluid, chemo-, mechano-, and photosensation, and sexual reproduction. The concept of ciliopathies has helped in advancing a unifying theory of cystic kidney diseases [22]. This theory states that the products of all genes that are mutated in cystic kidney diseases in humans, mice, or zebrafish are expressed in primary cilia or centrosomes of renal epithelial cells [22].

There are numerous disorders linked to basal body and/or cilia dysfunction, including polycystic kidney disease (PKD), primary ciliary dyskinesia (PCD), nephronophthisis (NPHP1–9) [23], Senior-Loken syndrome, Joubert syndrome, Meckel syndrome, oral-facial-digital syndrome, Alström syndrome and Bardet-Biedl syndrome [24]. These syndromes are typically associated with one or more of the symptoms like cystic kidneys [25], retinal degeneration and retinitis pigmentosa [16], *situs inversus *[26,27], anosmia [28], respiratory problems [26], infertility [26], hydrocephalus [29], other ailments like obesity, diabetes, liver fibrosis, hypertension, heart malformations, skeletal anomalies (e.g. polydactyly), cognitive impairment and developmental defects such as exencephaly [16,26].

Although the mechanisms of the cyst formation are not clearly understood, they are postulated to involve improper functioning of several pathways including cell proliferation, apoptosis, cell polarity, and fluid secretion [30]. Woo [31] has described apoptotic cells in glomeruli, cyst walls, and in both cystic and non-cystic tubules of the polycystic kidneys. Apoptotic loss of renal tissue may be associated with the progressive deterioration of renal function that occurs in patients with autosomal dominant polycystic kidney disease (ADPKD). There is evidence that genes involved in the regulation of cell proliferation, such as p53, c-fos, cyclin D1, and c-myc may be involved in the control of apoptosis [32]. Veis and colleagues have shown overexpression of c-myc in human ADPKD in association with increased levels of apoptosis and cell proliferation [33]. It is clear that pathogenesis of PKD is very complicated and involves multiple molecular pathways with overlapping, complementary, or opposing effects. There are several signalling pathways that have been implicated in ciliary function [34]. The dysregulation of mitogen-activated protein kinases (MAPKs) in the cyst epithelium of pcy (polycystic kidney disease) mice, carrying a missense mutation in NPHP3 [35], is a downstream consequence of disturbed renal monocilia function [36]. The proteins, implicated in the formation of renal cysts in tuberous sclerosis, have been found at the ciliary base. They form a complex that inhibits mTOR resulting in retarded cyst formation in rats with PKD [37]. Cilia-mediated signalling acts as a switch between canonical and non-canonical Wnt signalling pathways [34].

Rats or mice have been used as common model systems for the study of PKD. The Hannover rat, Han:Sprague-Dawley (SPRD)-*cy *rat is an accepted model for human PKD [38,39] and has been efficiently used since more than a decade. It is an autosomal dominant model for PKD resulting in cyst formation and slowly progressive chronic renal failure [39]. In the current investigation, we explore the transcriptional changes that occur in PKD and investigate if these changes could be related to miRNAs. We use a microarray-based approach to profile the transcriptional (mRNA) changes as well as changes in the miRNA expression patterns in PKD. We use the Han:SPRD cy/+ rat model [39] for our current investigation. Our results suggest several miRNAs may be involved in regulating the genetic switches in PKD. Furthermore we describe some newly detected miRNAs in the kidney.

## Methods

### Animals and physiological state

Inbred homozygous unaffected (+/+) and heterozygous affected (cy/+) Han:SPRD rats exhibiting PKD were investigated. The Han:SPRD-cy rats used in this study carry a dominant mutation that causes cystic kidneys and is an accepted model for human PKD [40]. After approximately 40 generations of inbreeding this substrain was registered as PKD/Mhm-cy inbred strain of rats: polycystic kidney diseases, Mannheim, Germany hereafter designated as PKD/Mhm. From each of the aforementioned PKD/Mhm (cy/+) and PKD/Mhm (+/+) animals, littermates were investigated. The animals were sacrificed by cervical dislocation at day 36. On the day of sacrifice, body weight was determined. Following dislocation, the left and right kidneys were immediately removed and weights were determined. Kidneys were preserved for histological analyses and the genotypes were confirmed. Histological analysis of pathogenesis was performed using hematoxylin-esosin (HE) staining of the kidney section (3 μm) followed by cyst grading under light microscope [41,42]; kidneys were graded on 1–5 scale as previously described [41,42].

We had ethical approval to carry out this work on animals by Regierungspraesidium Nordbaden and Internal Review Board.

### RNA isolation and Affymetrix microarray

Total RNA was extracted using TRIzol method according to manufacturer's protocol (Invitrogen Life Technologies). cDNA synthesis was performed using the SuperScript Choice System (Invitrogen Life Technologies, Invitrogen Corporation) according to manufacturer's protocol. Biotin-labelled cRNA was produced using ENZO BioArray High Yield RNA Transcript Labelling Kit. The standard protocol from Affymetrix (Santa Clara, CA) with 3.3 μL of cDNA was used for the *in vitro *transcription (IVT). Cleanup of the IVT product was done using CHROMA SPIN-100 columns (Clontech, USA). Spectrophotometric analysis was used for quantification of cRNA with acceptable A260/A280 ratio of 1.9 to 2.1. After that, the cRNA was fragmented using Affymetrix defined protocol. Labelled and fragmented cRNA was hybridized to Affymetrix Rat 230_2 microarrays for 16 hrs at 45°C using Affymetrix defined protocol. cRNA in the range of 15 μg was used for all the 12 microarrays. Microarrays were washed using an Affymetrix fluidics station 450 and stained initially with streptavidin/phycoerytherin. For each sample the signal was further enhanced by incubation with biotinylated goat anti-streptavidin followed by a second incubation with streptavidin/phycoerytherin, and a second round of intensities were measured. Microarrays were scanned with an Affymetrix scanner controlled by the Affymetrix Microarray Suite software. A total of 12 Affymetrix whole genome arrays (from 6 healthy and 6 diseased biological replicates) were performed.

### MicroRNA microarray

Locked Nucleic Acid (LNA) based miRNA hybridization assays were done as described by Castoldi et al. [43]. Briefly, 5 μg RNA was hybridized to a miRNA microarray (miChip v6.0), containing probes for ~300 miRNAs, based on LNA-modified and Tm-normalized oligonucleotide capture probes (miRCURY Array probes, Exiqon) spotted onto Codelink (GE Healthcare) in multiple replicates, and scanned using an Axon Scanner 4000B. Microarray images were analyzed using the Genepix Pro 4.0 software [43]. As for the Affymetrix whole genome arrays, 6 biologically replicated arrays, each for healthy and diseased animals (total of 12 chips) were performed.

### Statistical and bioinformatics analysis of microarray data

Microarray data obtained from Affymetrix chips were analyzed using SAS Micro-Array Solution version 1.3 (SAS, USA). Normalization of raw data was performed by fitting a mixed linear model across all arrays in the experiment. Log_2_-transformed scores of all spot measures were subjected to normalization. Identification of differentially expressed genes was carried out by Mixed Model Analysis (MMA) using ANOVA approach. A Bonferroni adjustment for multiple testing with α ≤ 0.05 was used to calculate the statistical significance threshold/cut-off [negative log_10 _(α)]. Custom CDFs version 8 (UniGene CDF files) [44] were used for annotations. Significantly up-regulated genes in PKD/Mhm (cy/+) animals were defined as those with >0 log-fold-changes in expression and down-regulated as those having <0 log-fold-changes in expression at p-value < 0.05 (adjusted for multiple testing).

The raw data from miRNA chips were also normalized and analyzed using the same modules in SAS Micro-Array Solution version 1.3, as described above. Significantly up-regulated miRNAs in PKD/Mhm (cy/+) animals were defined as those with >0 log-fold-changes in expression and down-regulated as those having <0 log-fold-changes in expression at p-value < 0.05 (adjusted for multiple testing).

Genes previously reported as targets for the significantly regulated miRNAs, and differentially regulated in PKD, were obtained from Argonaute [45]. In addition, targets for the significantly regulated miRNAs, in the genes differentially regulated in PKD, were predicted using two algorithms: TargetScan [46] and miRanda [47]. To identify the genes commonly predicted by both algorithms, results were intersected using a Perl script; the intersection of TargetScan and miRanda algorithms was used for further analysis.

### Identification of pathways possibly affected by miRNAs and their target genes in PKD

The pathways affected in PKD were determined for the significantly regulated genes from KEGG [48], GeneOntology (GO) [49], Biocarta  and the Molecular Signature database [50]. To check whether significantly regulated genes are overrepresented in a pathway in PKD or not, Fisher's exact test [51] without correction for multiple testing was used. Pathways showing p-value less than 0.05 were considered as significantly enriched.

### Reverse transcription and quantitative real-time PCR (qPCR) analysis of miRNAs

qPCR assays for miRNAs were performed using "TaqMan MicroRNA Assays" (Applied Biosystems, USA). The reverse transcriptase reactions were performed as per the manufacturer's protocol. Real-time PCR was also performed using a standard TaqMan PCR kit protocol on the Applied Biosystems 7000 Sequence Detection System. 6 biological replicates were used for analysis and all the reactions were run in quadruplicates. The comparative CT method for relative quantitation of gene expression was used to determine miRNA expression levels among the normal animals and PKD/Mhm (cy/+) animals according to manufacturer's protocol. miR-193a was used as an endogenous control to normalize the expression levels of targets. The miR-193a served as a good choice for endogenous control because its expression was almost uniform in all the samples on the miRNA-chips (0.99 ± 0.026) and it was not differentially regulated among the tested samples. miRNAs with ubiquitous and stable expression values are superior normalizers to other RNAs such as 5S rRNA, U6 snRNA or total RNA [52,53]. Consistency in expression of miR-193a across all the samples was evident in qPCR assays (mean CT-value 29.51 ± 0.19). The significance of differences in relative expression of miRNAs among the two groups was tested by One-way ANOVA method in SAS version 9.1.

## Results

### Pathological status of PKD/Mhm(cy/+) and healthy PKD/Mhm (+/+) animals

**Figure 1:**
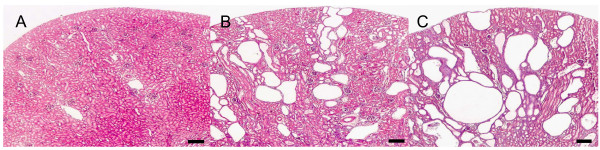
**Examples for different cyst gradings in 36 day old male PKD/Mhm rats**. At this age cyst grades of heterozygous (PKD/Mhm (cy/+)) affected kidneys are ranging between grade 3 (B) and 4(C), compared to a homozygous unaffected (healthy) kidney (A). Most of the animals are showing cyst grade 3. Hematoxylin/Eosin stained; Scale bar 250 μm.

We used 36 day-old PKD/Mhm(cy/+) and control (healthy) PKD/Mhm (+/+) animals to profile expression differences in m- and mi-RNAs. At this time the disease is still in the early stages. The Cy/+ Han:SPRD rat develops clinically detectable PKD by 8 weeks of age as evidenced by a doubling of kidney size and kidney failure compared with +/+ control rats. But disease symptoms in form of cysts can be observed using histological sectioning of the kidneys. Using HE staining followed by cyst counting, diseased kidneys can be grouped into 5 grades [41,42]. Grade 1 kidneys have occasionally small and medium-sized cysts and sometimes small accumulations of predominantly small cysts in up to four localizations per slide. Grade 2 kidneys have few regularly distributed small and medium-sized cysts with up to five medium-sized cysts per visual field, rarely large cysts (considerably larger than the size of the glomeruli), and cysts are not detected in every visual field. Grade 3 kidneys have several small and medium-sized cysts (up to 10 medium-sized cysts per visual field), few large cysts and cysts are in any visual field. A great number of small, medium-sized and large cysts with two or more large cysts in nearly any visual field and the occurrence of net-like structures that linked large cysts together are the characteristics of grade 4. In grade 5 there is practically no normal kidney tissue visible and the histology exhibits only large cysts and network-like structures. Six biological replicates graded 3–4 (Figure 1).

**Figure 2:**
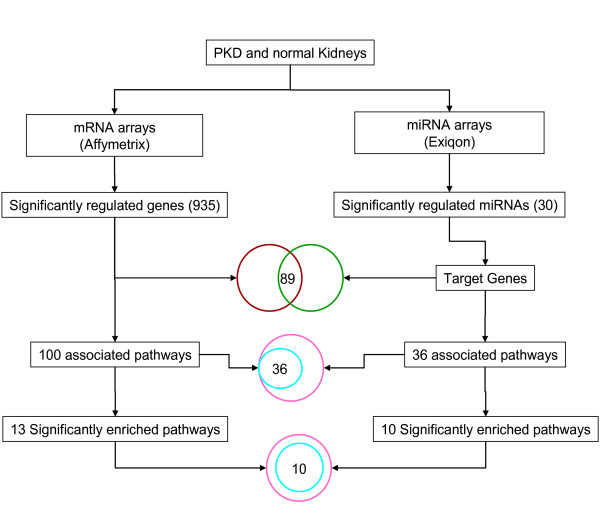
**Schematic representation of combinatorial approach identifying miRNAs and their targets**. mRNA and miRNA expression profilings were analysed by Mixed Model Analysis in SAS. 935 genes were differentially regulated on Affymetrix chips whereas 30 miRNAs were differentially changed on Exiqon chips. Target genes for differentially expressed miRNAs were obtained from Argonaute as well as TargetScan and miRanda and resulting genes were overlapped with differentially expressed mRNAs. Functionally related gene sets or pathways, determined for differentially expressed mRNAs and predicted target genes of differentially expressed miRNAs, were compared and the overlap gave 36 associated gene sets, out of which 10 were significantly enriched pathways.

In order to get insight into the genome-wide transcriptional rearrangements during PKD, we adapted a microarray based approach (Figure 2). We co-profiled the changes in transcript (mRNA) and miRNA expressions from the same sets of healthy and diseased kidney tissue using Affymetrix and Exiqon arrays for mRNA and miRNAs respectively.

### Profiling miRNA expression change during PKD

**Figure 3:**
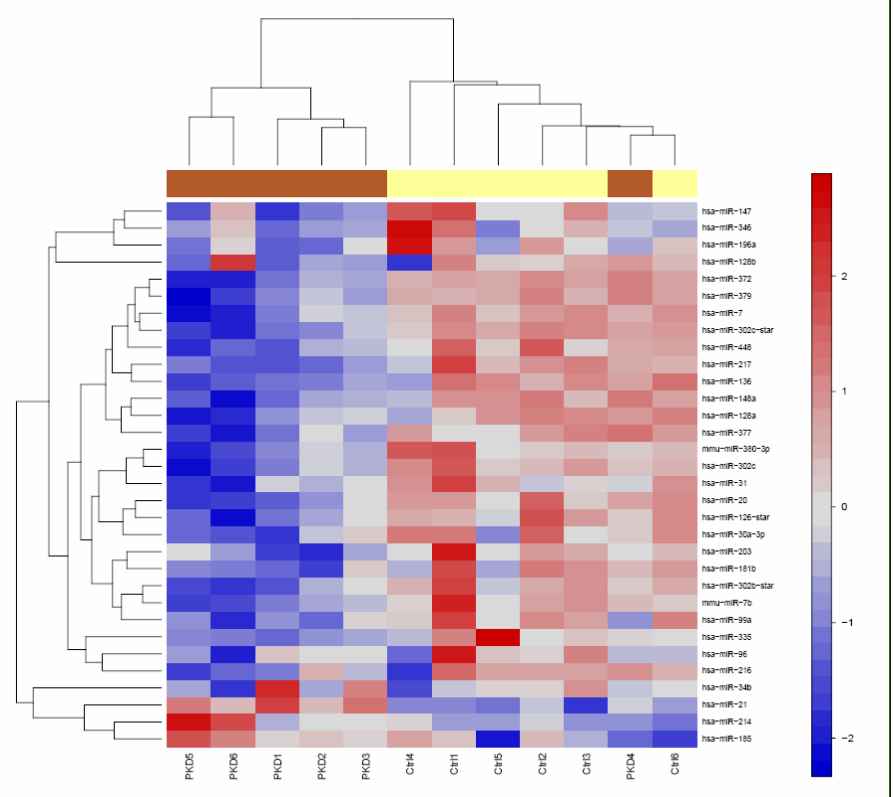
**Differential expression of miRNA in PKD and control (Ctrl) animals**. Heatmap was produced using simultaneous clustering of rows and columns of the data matrix using complete linkage algorithm and a euclidean distance metric. Prior to clustering, values were transformed to zero (row-wise) mean and unit (row-wise) variance. The miRNA clustering tree is shown on the left and the sample clustering tree is shown on the top. The samples are clustering broadly into two groups, control (Ctrl) and PKD. The color scale shown at the right illustrates the relative expression level of the indicated miRNA across all samples: red denotes expression > 0 and blue denotes an expression < 0. miRNAs shown here are from miRNA microarrays.

**Figure 4:**
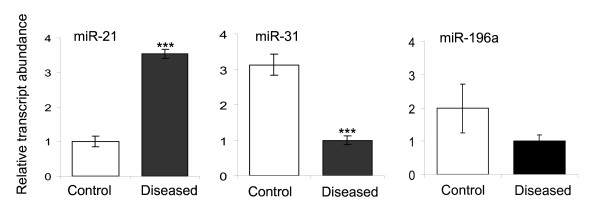
**qPCR analysis of miRNAs in PKD**. Expression of 3 miRNAs (miR-21, 31 and 196a) significantly regualted on miRNA microarray was verified using qPCR assays. *** significantly different at P < 0.001.

In order to investigate the changes in miRNA profiles, we examined the expression of miRNAs between kidneys of PKD/Mhm (cy/+) and control PKD/Mhm (+/+) animals using LNA-based miRNA-chips [43]. miRNAs are highly conserved between human, mouse and rat [54], therefore, above said chips could be effectively used to profile miRNA in our rat model. We identified 30 differentially regulated miRNAs (Table 1), distributed in 21 different families, at a cut-off ≥ 1.7 (negative log_10 _(p-value ≤ 0.05, after Bonferroni's correction for multiple testing)). Out of 30 miRNAs, 29 were down-regulated in PKD/Mhm (cy/+) animals and only one miRNA, miR-21, was up-regulated. Cluster analysis of chips showed that miRNA-microarray was able to distinguish 83.3% diseased and healthy animals (Figure 3). Interestingly, the expressions of miR-31 and miR-217 have not been previously reported in kidney. We verified the changes in the expression patterns of some of these miRNAs using quantitative real-time PCR (qPCR; Figure 4). We verified the expression changes of the only up-regulated miRNA, miR-21: in the qPCR assays, it was 3.5 fold up-regulated in the kidney of diseased animals, compared to healthy ones. In line with the expression on the miRNA-arrays, in qPCR analysis (Figure 4), miR-31 was 3.15 fold down regulated in diseased samples compared to healthy tissue.

**Table 1:** Significantly regulated miRNAs on Exiqon chips from SAS analysis

**Name of miRNAs on chip**	miRNA name in rat*	**Lsmean animal PKD**	**Lsmean animal Control**	**Log Fold Change PKD_Ctrl**	**-log10(p-value) for log fold change of PKD_Ctrl**
hsa-miR-21	rno-miR-21	0.80	0.24	0.56	4.50
hsa-miR-302c-star		-0.63	-0.33	-0.30	3.32
hsa-miR-31	rno-mir-31	-0.47	-0.13	-0.34	3.22
hsa-miR-302c		-0.76	-0.30	-0.46	3.22
hsa-miR-217	rno-mir-217	-0.74	-0.41	-0.33	3.09
mmu-miR-34b	rno-mir-34b	-0.59	-0.35	-0.24	3.00
hsa-miR-126-star	rno-mir-126-star	-0.32	-0.07	-0.25	2.98
hsa-miR-7	rno-mir-7	-0.60	-0.23	-0.37	2.92
hsa-miR-128b	rno-mir-128	-0.69	-0.37	-0.32	2.92
hsa-miR-302b-star		-0.53	-0.27	-0.26	2.76
hsa-miR-136	rno-mir-136	-0.70	-0.37	-0.33	2.75
hsa-miR-99a	rno-mir-99a	-0.40	-0.14	-0.26	2.68
hsa-miR-448	rno-mir-448	-0.55	-0.27	-0.28	2.64
mmu-miR-380-3p	rno-mir-380	-0.63	-0.12	-0.51	2.62
hsa-miR-20	rno-mir-20	-0.31	-0.12	-0.19	2.58
hsa-miR-96	rno-mir-96	-0.65	-0.40	-0.26	2.57
hsa-miR-372		-0.75	-0.46	-0.29	2.55
mmu-miR-7b	rno-mir-7b	-0.46	-0.13	-0.34	2.48
hsa-miR-379	rno-mir-379	-0.74	-0.40	-0.34	2.47
hsa-miR-203	rno-mir-203	-0.50	-0.23	-0.27	2.34
hsa-miR-147	rno-mir-147	-0.66	-0.42	-0.23	2.16
hsa-miR-196a	rno-mir-196a	0.13	0.38	-0.25	2.10
hsa-miR-335	rno-mir-335	-0.72	-0.21	-0.51	1.94
hsa-miR-216	rno-mir-216	-0.70	-0.41	-0.29	1.94
hsa-miR-128a	rno-mir-128	-0.65	-0.32	-0.33	1.92
hsa-miR-30a-3p	rno-mir-30a	-0.15	0.03	-0.18	1.90
hsa-miR-148a		-0.39	-0.27	-0.12	1.85
hsa-miR-181b	rno-mir-181b	-0.35	-0.14	-0.20	1.80
hsa-miR-346	rno-mir-346	-0.69	-0.39	-0.30	1.78
hsa-miR-377	rno-mir-377	-0.58	-0.35	-0.23	1.75

*miRNA probes present on Exiqon chip start with name "hsa-" and "mmu-" as shown in column 1. The miRNAs listed here are conserved in "Rat", shown in column 2.

### Microarray analysis of genes involved in PKD

To study the genetic regulation in PKD, we performed gene expression profiling study using Affymetrix chips (RAE230_2) on PKD/Mhm (cy/+) and control, PKD/Mhm (+/+), rats. The arrays were analyzed by SAS Micro-Array Solution version 1.3. A number of 1740 probe sets, according to custom CDF version 8 [44], corresponding to 935 genes were found to be significantly regulated at a cut-off (negative log_10_) of 5.5584 (Additional file 1). Several genes strongly associated with PKD such as Clusterin [55,56], Vimentin [56], Timp [56], Bcl2 [57], several members of MAPK signaling [58] etc. were strongly differentially regulated in expected orientations, showing the reliability of mRNA expression profiles. Table 2 shows the top 25 significantly up/down-regulated genes according to the negative log_10 _of p-values.

**Table 2:** Top 25 up-regulated and 25 down-regulated genes on Affymetrix chips from SAS analysis

**Affymetrix_ID**	**UniGene ID**	**Gene_Symbol**	**Log_fold_change (>0 = up-regulated; <0 = down-regulated)**	**-log10(p-value) for Estimate of diseased_healthy**
**1367581_a_at, 1396689_at**	**Rn.8871**	**Spp1**	**2.2450**	**41.2288**
**1378015_at**	**Rn.39658**	**RGD:1311996**	**1.9669**	**35.0162**
**1367784_a_at**	**Rn.1780**	**Clu**	**3.3779**	**29.5397**
**1367913_at**	**Rn.105938**	**Cygb**	**1.4106**	**28.3857**
**1381343_at, 1383222_at**	**Rn.1708**	**LOC257646**	**0.8336**	**22.6443**
**1386996_at**	**Rn.103179**	**RGD:628855**	**0.7756**	**22.4535**
**1388114_at**	**Rn.103179**	**Mrlcb**	**0.7756**	**22.4535**
**1388745_at**	**Rn.54039**	**RGD:1304636**	**0.7278**	**22.2871**
**1370992_a_at, 1371258_at**	**Rn.98846**	**Fga**	**1.0091**	**21.9972**
**1370186_at**	**Rn.13686**	**Psmb9**	**0.9171**	**21.6556**
**1398347_at**	**Rn.16368**	**RGD:1312008**	**1.1720**	**21.2353**
**1368504_at, 1398816_at**	**Rn.40177**	**Lamp1**	**0.4187**	**20.6298**
**1368168_at**	**Rn.16933**	**RGD:620889**	**2.1632**	**20.4333**
**1387391_at**	**Rn.10089**	**Cdkn1a**	**0.4408**	**20.1517**
**1373204_at**	**Rn.17071**	**RGD1310725_predicted**	**1.1134**	**18.9746**
**1369956_at**	**Rn.19927**	**RGD:620570**	**0.5636**	**18.7804**
**1385625_at, 1393240_at**	**Rn.16151**	**Efemp2**	**1.0126**	**18.3754**
**1368207_at**	**Rn.24997**	**Fxyd5**	**0.7122**	**17.9030**
**1371244_at**	**Rn.2498**	**RGD:1307758**	**0.5877**	**17.6513**
**1368160_at**	**Rn.34026**	**Igfbp1**	**1.0420**	**17.1419**
**1368821_at, 1368822_at, 1371331_at, 1394119_at**	**Rn.95652**	**Fstl1**	**0.7759**	**17.1139**
**1389189_at, 1396539_at, 1398294_at**	**Rn.6401**	**Actn1**	**1.4167**	**16.9389**
**1367712_at**	**Rn.25754**	**Timp1**	**1.3548**	**16.9170**
**1387892_at**	**Rn.2458**	**Tubb5**	**1.0106**	**16.8147**
**1367823_at, 1375144_at, 1386940_at, 1388312_at**	**Rn.10161**	**Timp2**	**0.2867**	**16.7147**
1369400_a_at, 1388044_at, 1388063_a_at, 1398320_at	Rn.44844	Pfkfb2	-0.2293	20.3237
1370268_at	Rn.44291	Kcna5	-0.2714	17.3640
1369178_a_at, 1385148_at	Rn.91176	P2rx1	-0.2108	5.5590
1378419_at	Rn.87496	LOC474169	-0.3129	5.5606
1369279_at	Rn.81185	Dhrs9	-0.2163	5.5612
1388051_at	Rn.81026	Slc26a3	-0.3637	5.5618
1368695_at	Rn.11151	C4bp-ps1	-0.2888	5.5684
1369341_a_at	Rn.7033	Acvrinp1	-0.1886	5.5718
1394129_at, 1396858_at	Rn.7033	Acvrip1	-0.1886	5.5718
1369358_a_at	Rn.37430	Hap1	-0.1699	5.5727
1387320_a_at	Rn.89629	RGD:708527	-0.1887	5.5781
1367571_a_at, 1371206_a_at, 1398322_at	Rn.118681	Igf2	-0.1007	5.5784
1371564_at	Rn.103171	RGD:735157	-0.1572	5.5789
1387292_s_at, 1387991_at	Rn.80837	Capn8	-0.2614	5.5793
1369253_at	Rn.90021	Kremen	-0.2313	5.5831
1369240_a_at	Rn.10096	Avpr1b	-0.2056	5.5835
1382417_at	Rn.90967	RGD:1309306	-0.1498	5.5848
1369552_at	Rn.81187	Samsn1	-0.2144	5.5849
1368763_at	Rn.10652	Il3	-0.2808	5.5851
1369444_at	Rn.21402	Cyp19a1	-0.3457	5.5866
1387582_a_at	Rn.81203	Pde7b	-0.2523	5.5870
1375494_a_at, 1387409_x_at	Rn.10263	Nlgn3	-0.1551	5.5897
1377146_at	Rn.18675	RGD:621647	-0.2512	5.5908
1387615_at	Rn.44391	RGD:621843	-0.2146	5.5965
1387065_at, 1395839_at	Rn.37434	Plcd4	-0.2386	5.5978

In order to reveal the functional meaning of differentially regulated genes, we analyzed the biological pathways these genes may be involved in. We used a curated list of 162 gene sets (pathways) (Additional file 2) corresponding to 1335 genes. They had been obtained from KEGG, MSigDB, GO and Biocarta  to identify pathways of significantly regulated genes in [48-50]. Out of 935 significantly regulated genes, only 233 genes were found in this list. These 233 genes were involved in 100 pathways (as shown in Figure 2), which were further categorized into 24 functional groups, based on KEGG terminology (Additional file 3, Figure 5). Over-representation analysis (ORA) examines the genes that meet a selection criterion and determines if there are gene sets which are statistically over-represented. An ORA of these pathways showed that 13 of them were significantly enriched (P < 0.05) in the PKD (cy/+) animals (as shown in Figure 2). Of those, major signal transduction signalling pathways previously described [58-60] as involved in various aspects of PKD, such as TGF-β, mTOR signalling pathway, MAPK, calcium signalling pathway, Wnt and JAK/STAT signalling pathways, were regulated.

**Figure 5:**
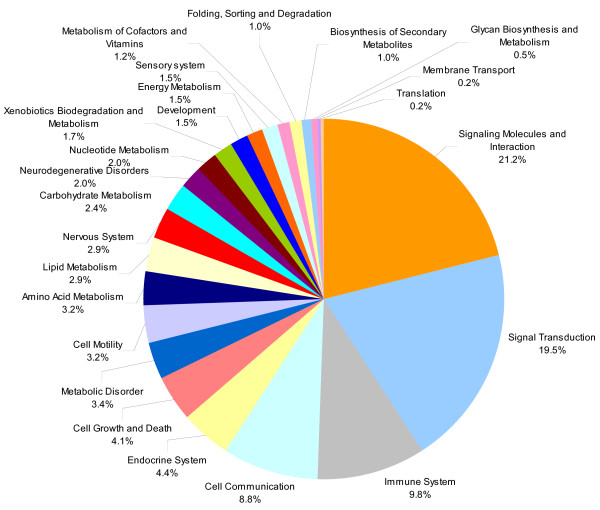
**Overrepresented miRNA regulatory pathways in PKD**. Fisher's exact test was used to identify significant enrichment for pathway annotations among predicted targets of dysregulated miRNAs and differentially expressed mRNAs in PKD. Pathways (from KEGG, MSigDB, Biocarta, GO) have been grouped in larger functional categories according to the KEGG annotation. The pie-chart shows 22 functional categories and each pie represents a functional category with an overrepresentation of regulatory pathways of miRNA targets as well as mRNAs. Only pathways overlapped from the functional patterns of predicted miRNA targets and differentially expressed mRNAs are shown.

### Identifying miRNA-target interaction

**Figure 6:**
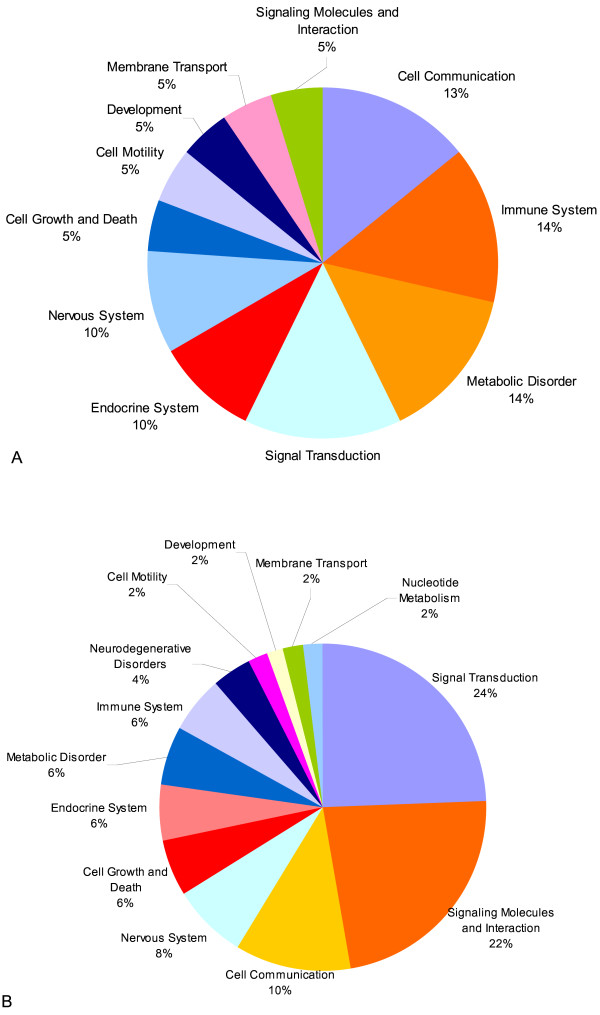
**Overrepresented miRNA regulatory pathways in PKD (for target genes obtained from Argonaute)**. **6A**. The pie-chart shows overrepresented pathways for the target genes obtained from Argonaute. Each pie denotes an overrepresentation of regulatory pathways of predicted targets of dysregulated miRNA as well as differentially expressed mRNAs in PKD; and also the percentage of genes overrepresented in the pathway. **6B**. Similarly here the pie-chart shows overrepresented pathways for the predicted miRNA targets obtained from TargetScan as well as miRanda.

Our miRNA-micorarray profiling revealed changes in miRNA expression pattern, indicating that miRNAs play a role in regulating the gene expression during PKD. We mapped the possible targets of these differentially regulated miRNAs to differentially regulated genes (935, obtained from our mRNA expression experiment). We first looked for already reported targets by comparing the list of differentially regulated miRNAs and the genes to the available miRNA-target interactions present in the Argonaute database [45]. A total of 25 genes are reported as targets of 15 miRNAs (Table 3a). But none of these miRNA-target interactions were reported in PKD. Out of these 25 genes, 11 genes are known to affect 23 pathways that were grouped into 11 functional categories (Figure 6a). Five pathways, namely GnRH signaling pathway, MAPK signaling pathway, Long term depression, Calcium signaling pathway and Neuroactive ligand receptor interaction were significantly enriched in PKD animals, as revealed by ORA.

**Table 3:** miRNAs and their targets

a: miRNAs and their previously reported targets (from Argonaute database). miRNAs and their corresponding targets are both differentially regulated during PKD.		
**miRNA**	**Target Genes**	**Pathways**

miR-128	ABCB9, BTG1, DSCR1, RASD1	ABC transporters General
miR-136	GRN, PPP1R9B	
miR-147	HOXA1, PTGFRN	
miR-148	EGR3, SCN3A	
miR-181b	IGF1R, NKX6-1	Adherens junction, Maturity onset diabetes of the, Focal adhesion, **Long term depression
miR-196a	ABCB9, CPB2, IRS1, MAPK10	ABC transporters General, Complement and coagulation cas, Adipocytokine signaling pathwa, Insulin signaling pathway, Type II diabetes mellitus, Fc epsilon RI signaling pathwa, Focal adhesion, **GnRH signaling pathway, **MAPK signaling pathway, Toll like receptor signaling p, Wnt signaling pathway
miR-203	SARA1	
miR-20	BTG1, SARA1, YWHAB	Cell cycle
miR-21	TPM1	
mir-216	GNAZ	**Long term depression
miR-217	RHOA	Adherens junction, Axon guidance, Focal adhesion, Leukocyte transendothelial mig, Regulation of actin cytoskelet, TGF beta signaling pathway, T cell receptor signaling path, Tight junction, Wnt signaling pathway
miR-31	ATP2B2, DNM1L, EGR3, PPP1R9B, YWHAB	**Calcium signaling pathway, Cell cycle
miR-7	SLC23A2	
miR-7b	HRH3, NCDN, SLC23A2	**Neuroactive ligand receptor in

b: miRNAs and their targets (from TargetScan and miRanda). miRNAs and their corresponding targets are both differentially regulated during PKD.		

**miRNA**	**Target Genes**	**Pathways**

miR-128	ABCB9, BTG2, CACNB2, CNR1, COL3A1, GLRA2, IRS1, NEK6, PDE7B, PTPN5, SV2A, SYT4, YWHAB, NR5A2, NTRK3	ABC transporters General, **MAPK signaling pathway, **Neuroactive ligand receptor in, **Cell Communication, **ECM receptor interaction, Focal adhesion, Adipocytokine signaling pathwa, Insulin signaling pathway, Type II diabetes mellitus, Purine metabolism, Cell cycle, Maturity onset diabetes of the
miR-136	KCNH7	
miR-148	OTX1, YWHAB	Cell cycle
miR-181b	ADAMTS1, ATP2B2, CACNB2, CDH13, CNR1, DUSP6, EGR3, EPHA7, GRIK2, GRM5, GRM7, HOXA1, MMP14	**Calcium signaling pathway, **MAPK signaling pathway, **Neuroactive ligand receptor in, Axon guidance, Gap junction, **Long term depression, Long term potentiation, **GnRH signaling pathway
miR-196a	ABCB9, COL3A1, EPHA7, GAS7, OTX1	ABC transporters General, **Cell Communication, **ECM receptor interaction, Focal adhesion, Axon guidance
miR-203	TGFB3	Cell cycle, **MAPK signaling pathway, TGF beta signaling pathway
miR-21	BTG2	
miR-217	CLU, FN1, GRIK2, KCNH5, NR4A2, RAP1B	**Cell Communication, **ECM receptor interaction, Focal adhesion, Regulation of actin cytoskelet, **Neuroactive ligand receptor in, Leukocyte transendothelial mig, Long term potentiation, **MAPK signaling pathway
miR-302b*	ATP2B2	**Calcium signaling pathway
miR-302c	ATP2B2, CNR1, SNF1LK	**Calcium signaling pathway, **Neuroactive ligand receptor in
miR-302c*	ATP2B2, SNF1LK	**Calcium signaling pathway
miR-30a-3p	CCKBR, COLEC12, EPHA7, GRM7, JUN, KCNAB1, LIN7A, MAMDC1, PRRX1	**Calcium signaling pathway, **Neuroactive ligand receptor in, Axon guidance, B cell receptor signaling path, Focal adhesion, **GnRH signaling pathway, **MAPK signaling pathway, T cell receptor signaling path, Toll like receptor signaling p, Wnt signaling pathway
miR-346	GRM7, LTBP2	**Neuroactive ligand receptor in
miR-372	ATP2B2, NR4A2, SNF1LK, RET, SLC11A2	**Calcium signaling pathway
miR-448	COLEC12, DNM3, GRIK2, IGF1R, IL12B, KCNH7, KCNIP4, SCN3A, SCN5A	**Neuroactive ligand receptor in, Adherens junction, Focal adhesion, **Long term depression, **Cytokine cytokine receptor int, **Jak STAT signaling pathway, Toll like receptor signaling p, **Type I diabetes mellitus
miR-34b	BTG2, CALCR, DLL1, MYRIP	**Neuroactive ligand receptor in, Notch signaling pathway
miR-380-3p	BCL2L1	Amyotrophic lateral sclerosis, Apoptosis, **Jak STAT signaling pathway, Neurodegenerative Disorders
miR-7b	EGR3	

**Significantly overrepresented pathways from ORA

Secondly, we mapped the differentially regulated genes for miRNA targets for the differentially regulated miRNAs using two tools, TargetScan [46] and miRanda [47]. Only commonly predicted targets for different miRNAs were taken for further analysis. A number of 65 genes were identified as miRNA targets, common from TargetScan as well as miRanda (Table 3b), out of which 31 genes participate in regulating 33 pathways, which could further be grouped into 13 functional categories (Figure 6b). The intersection of TargetScan and miRanda results gave target genes for 20 miRNAs out of 30 differentially regulated miRNAs. This shows that available prediction tools are not yet fully optimal. Altogether, we obtained 89 target genes differentially expressed on Affymetrix chips (Figure 2). These were associated with 36 pathways, out of which 10 were significantly enriched by ORA (Table 3a and 3b; Figure 2).

miRNAs are generally thought to negatively regulate the expression of their targets by mRNA degradation or by repressing translation [61]. So if a given miRNA is down-regulated, the expression of its target is expected to be up-regulated. In light of this mode of miRNA action, we next scrutinized the relationship of the expression patterns of the differentially regulated miRNA and their targets identified in the above exercise. Eleven targets for 11 miRNAs had expression patterns in opposite direction (Table 4), indicating that these relationships may be functional miRNA-target combinations in PKD. We were unable to find a direct, down-regulated target, for miR-21.

**Table 4:** Selected inverse miRNA-target relation identified

**miRNA**	**Target Genes**	**Pathways**	**Identified by**	**Log fold change miRNA**	**Log fold change gene**
**hsa-miR-128a**	*COL3A1*	**Cell Communication, **ECM receptor interaction, Focal adhesion	TargetScan miRanda	**-0.32881**	*1.36067*
**hsa-miR-128a**	*YWHAB*	Cell cycle	TargetScan miRanda	**-0.32881**	*0.39416*
**hsa-miR-128b**	*COL3A1*	**Cell Communication, **ECM receptor interaction, Focal adhesion	TargetScan miRanda	**-0.32151**	*1.36067*
**hsa-miR-128b**	*YWHAB*	Cell cycle	TargetScan miRanda	**-0.32151**	*0.39416*
**hsa-miR-148a**	*YWHAB*	Cell cycle	TargetScan miRanda	**-0.11883**	*0.39416*
**hsa-miR-181b**	*IGF1R*	Adherens junction, Focal adhesion, **Long term depression	Argonaute	**-0.20269**	*0.39429*
**hsa-miR-181b**	*MMP14*	**GnRH signaling pathway	TargetScan miRanda	**-0.20269**	*0.63365*
**hsa-miR-181b**	*DUSP6*	**MAPK signaling pathway	TargetScan miRanda	**-0.20269**	*0.66781*
**hsa-miR-181b**	*GRIK2*	**Neuroactive ligand receptor in	TargetScan miRanda	**-0.20269**	*0.54227*
**hsa-miR-196a**	*COL3A1*	**Cell Communication, **ECM receptor interaction, Focal adhesion	TargetScan miRanda	**-0.24597**	*1.36067*
**hsa-miR-203**	*TGFB3*	Cell cycle, **MAPK signaling pathway, TGF beta signaling pathway	TargetScan miRanda	**-0.26753**	*0.38630*
**hsa-miR-20a**	*YWHAB*	Cell cycle	Argonaute	**-0.18897**	*0.39416*
**hsa-miR-217**	*RHOA*	Adherens junction, Axon guidance, Focal adhesion, Leukocyte transendothelial mig, Regulation of actin cytoskelet, T cell receptor signaling path, TGF beta signaling pathway, Tight junction, Wnt signaling pathway	Argonaute	**-0.32869**	*0.56512*
**hsa-miR-217**	*FN1*	**Cell Communication, **ECM receptor interaction, Focal adhesion, Regulation of actin cytoskelet	TargetScan miRanda	**-0.32869**	*1.15979*
**hsa-miR-217**	*RAP1B*	Focal adhesion, Leukocyte transendothelial mig, Long term potentiation, **MAPK signaling pathway	TargetScan miRanda	**-0.32869**	*0.40226*
**hsa-miR-217**	*GRIK2*	**Neuroactive ligand receptor in	TargetScan miRanda	**-0.32869**	*0.54227*
**hsa-miR-30a-3p**	*JUN*	B cell receptor signaling path, Focal adhesion, **GnRH signaling pathway, **MAPK signaling pathway, T cell receptor signaling path, Toll like receptor signaling p, Wnt signaling pathway	TargetScan miRanda	**-0.18488**	*0.64628*
**hsa-miR-31**	*YWHAB*	Cell cycle	Argonaute	**-0.33805**	*0.39416*
**hsa-miR-448**	*IGF1R*	Adherens junction, Focal adhesion, **Long term depression	TargetScan miRanda	**-0.28453**	*0.39429*
**hsa-miR-448**	*GRIK2*	**Neuroactive ligand receptor in	TargetScan miRanda	**-0.28453**	*0.54227*

## Discussion

In the current investigation, we explore the possible involvement of miRNAs in PKD. In a well characterized rat model system (Han:SPRD cy/+ rat model; [39]), we used a combinatorial approach involving data mining and microarray analysis, to profile the miRNAs involved in PKD. In parallel to the miRNA expression profiling, we also describe the changes in mRNA transcript patterns in PKD using genome-wide Affymetrix arrays. Whereas, the gene expression arrays e.g. Affymetrix arrays have already established themselves for measuring large-scale differential regulation of mRNAs, microarrays have recently been developed to measure miRNA expression changes accurately [43,62]. We used arrays based on LNA technology [43]. The cluster analysis of the control and PKD miRNA-microarrays showed tight clustering PKD samples (Figure 3). Few studies have appeared showing the use of proteomics based approaches for identifying specific miRNA targets [63-66]. Several of these studies also applied mRNA-expression arrays to derive correlations between the protein- and messenger- turndown. Over-expressing miRNAs resulted in repressed targets, both at protein and messenger levels: how much both processes contribute to down-regulation of targets depends on individual miRNA-mRNA pairs [65]. These studies show that mRNA profiling are indeed valuable tools for studying mRNA-miRNA interaction, although additional information on fine-tuning of proteome-expression may be obtained by including proteomics approaches.

Our study, profiling the changes in the mRNA transcripts in PKD, revealed large-scale changes. Although some transcription factors, such as SP1 [67], JUN and c-myc [68] have been implicated to play a role in PKD, the magnitude of transcriptional changes (>900 genes) suggest involvement of other regulators. Moreover, the regulatory basis of changes in the expression of transcription factors involved in PKD remains poorly understood. On the other hand, miRNAs have emerged recently as key regulators of gene expression in many developmental [69,70] and disease events [71,72]. Our miRNA study shows that changes in gene expression during PKD also involve miRNAs.

When we compared the mRNA and miRNA profiles, differentially regulated in PKD, with Argonaute (a comprehensive database on miRNAs; [45,71]), there were few genes reported as miRNA target like tropomyosin 1, alpha (TPM1) as a target of miR-21, the beta polypeptide of tyrosine 3-monooxygenase/tryptophan 5-monooxygenase activation protein (YWHAB), regulatory subunit 9B of protein phosphatase 1 (PPP1R9B), early growth response 3 (EGR3) and dynamin 1-like (DNM1L) as targets of miR-31, plysia ras-related homolog A2 (RHOA) as targets of miR-217, etc. (Table 3a). But none of these genes had previously been associated with PKD. Over-representation analysis showed the enrichment of five pathways namely, GnRH signaling pathway, MAPK signaling pathway, Long term depression, Calcium signaling pathway and Neuroactive ligand receptor interaction. The comparison of differentially regulated genes/miRNAs to Argonaute database indicates that previously known miRNA-target interactions (Table 3a) could play important roles in PKD.

Parallel profiling of the transcripts as well as miRNAs on the same set of samples gives us insight into potential interactions between miRNAs and differentially expressed targets in PKD. This parallel profiling allowed us to scrutinize for the negative expression patterns for the miRNA-target relation at a given time point (Table 4). A negative relationship of the expression patterns between miRNA and its target mRNA is an important parameter for determining their interactions because miRNA, in general, are regarded as negative regulators of their targets.

Microarray analysis of miRNAs revealed down regulation of 29 out of 30 differentially regulated miRNAs, which corresponded to increased expression of several genes related to pathways upregulated during PKD. To further uncover the functional correlation between differentially expressed mRNAs and miRNAs, we determined functionally related gene sets, or pathways. The differentially regulated mRNAs were associated with 24 functional categories, which included several enriched pathways important to renal diseases. Previous studies of cystic kidneys implicated several pathways thought to contribute to the pathogenesis of renal cysts' formation like mTOR signalling, MAPK (Mitogen-Activated Protein Kinase) signalling, Wnt signalling, and the TGF-β (Transforming Growth Factor-β) pathway.

MAPKs play important roles in the cell by transmitting extracellular signals from the cell membrane to the nucleus [73]. MAPKs are activated by various stimuli, influencing cell proliferation, differentiation, and apoptosis. Aberrant regulation of MAPKs and other signalling pathways has been reported to be consistent with altered regulation of cell proliferation and differentiation observed in renal cystic disease [58]. Sustained activation of MAPKs in kidney epithelial cells inhibits normal epithelial phenotype and formation of adherens junctions [74]. The expression of genes involved in MAPK signalling pathway like MAP3K1, JUN, and MYC was significantly higher in the PKD animals, indicating the activation of MAPK signalling pathway.

Wnt signalling is essential for renal development. Recent research has revealed an unexpected intersection between Wnt signalling and PKD [59]. It has been reported that canonical Wnt signalling seemed mandatory for early renal development, but persistent β-catenin signalling seemed to trigger cyst formation at later developmental stages [59]. Components of the Wnt signalling pathway like FZD2, JUN, MYC, and RHOA were significantly upregulated in our PKD animals.

TGF-β promotes renal cell hypertrophy and stimulates extracellular matrix accumulation in several renal diseases, including diabetic nephropathy [75] and PKD [76]. It activates the inhibitors of the proteases e.g. tissue inhibitors of metalloproteinases and plasminogen activator inhibitor 1 [77]. TGF-β 1, TGF-β 2 and TGF-β 3 were consistently upregulated in PKD-affected animals. The expression of extracellular matrix components, such as collagen triple helix repeat containing 1 (CTHRC1), and fibronectin 1 (FN1) was significantly higher in animals with PKD. One of the integrins, integrin beta 1 (ITGB1) was upregulated. Therefore, there was a clear activation of the TGF-β signaling pathway in the PKD animals with a significant increase of the synthesis of the extracellular matrix components and inhibition of the proteases that digest this matrix. Recently Kato et al. showed that miRNA miR-192 has been associated with TGF-β pathway in diabetic kidney glomeruli [78]. Its implication in PKD has not been shown, though.

We could not find negative co-relations between the predicted targets of the only up-regulated miRNA, miR-21, whose expression was verified by qPCR analysis too. All the predicted targets were up-regulated. It should be noted that alternate action mechanisms for miRNAs also exist. The action of miRNAs may not to be reflected on the level of their target mRNAs as they are believed to block or attenuate further translation of mRNAs to protein. In such conditions, miRNAs will exert their regulatory role on the level of translation. Moreover, a new mode of action where the miRNAs act as positive regulators has also been defined recently [7]. Depending upon the state of a cell, a miRNA can act as positive or negative regulator [7].

The specific cellular pathways that were found to be associated with dysregulated miRNAs or differentially expressed transcripts in PKD can be used to shape some initial hypotheses on how alteration of miRNA expression may be directly involved in the disease. Furthermore, the functional correlation between the differentially expressed mRNAs and miRNAs in PKD revealed a tight posttranscriptional regulation network at both mRNA and protein level.

## Conclusion

While this work establishes a multi-tiered approach for investigation of miRNA mode of genetic regulations of PKD, the results await further investigation. PKD is an important disease as 8–10% of all patients on renal replacement therapy including haemodialysis or transplantation, suffer from PKD [79]. The molecular components have just started to become apparent, and we add another layer of regulators, namely miRNAs. We predict that several of the differentially regulated genes are miRNA targets and miR-21, miR-31, miR-128, miR-147 and miR-217 may be the important players in such interaction. It is interesting to note that miR-31 and miR-217 have not been previously reported in kidney. miR-21 has been reported in kidney [45] but its function has yet not been established. It has been speculated in previous studies that a single miRNA could target more than hundred genes and one gene could be the target of several miRNAs [61,80]. Knockout and over-expression studies will provide further insight into the regulatory interaction between these miRNAs and their targets in order to properly understand PKD development and design new therapeutic measures.

## Authors' contributions

PP carried out the study, analysed and interpreted the data and drafted the manuscript. BB participated in the design of the study and drafting the manuscript. PKS participated in analysis of data. AB helped in animal experiments. SB helped in animal experiments. HJG participated in revising the manuscript critically for important intellectual content. NG designed the study, participated in drafting the manuscript and has given final approval of the version to be published.

## Supplementary Material

Additional file 1 **Significantly regulated mRNAs.** This table shows significantly regulated mRNAs on the Affymetrix chips obtained from SAS analysis. Click here for file

Additional file 2 **Curated pathway list. **This table shows the curated pathways obtained from KEGG, Biocarta, MSigDB and GO. Click here for file

Additional file 3 **Categories of pathways.** This table shows the pathway categories based on KEGG annotation for significantly regulated genes on Affymetrix chips. Click here for file
